# Developing a production process for influenza VLPs: a comparison between HEK 293SF and Sf9 production platforms

**DOI:** 10.1186/1753-6561-7-S6-P22

**Published:** 2013-12-04

**Authors:** Christine M Thompson, Emma Petiot, Marc G Aucoin, Olivier Henry, Amine A Kamen

**Affiliations:** 1 Human Health Therapeutics, Vaccine Program, NRC, Montréal, Québec, H4P 2R2, Canada; 2 Department of Chemical Engineering, École Polytechnique de Montréal, Montréal, Québec, H3C 3A7, Canada; 3 Department of Chemical Engineering, University of Waterloo, Waterloo, Ontario, N2L 3G1, Canada

## Background

Influenza virus-like particle (VLP) vaccines are one of the most promising approaches to respond to the constant threat of the emergence of pandemic strains, as they possess the potential for higher production capabilities compared to traditional vaccines made in egg-based technology. VLPs are particles produced in cell culture utilizing recombinant protein technology composed of viral antigens that are able to elicit an immune response but lack viral genetic material. Thus far, influenza VLPs have been produced in mammalian, insect and plant based platforms [1], with production in insect cells being the most explored. Baculovirus with mammalian promoters (Bacmam) have been shown to efficiently transduce mammalian cells and further express genes but are unable to replicate, efficiently repressing baculovirus (BV) production that leads to contamination downstream [2]. Influenza VLP production was performed in HEK 293SF cells using the Bacmam gene delivery system. The proposed system was assessed for its ability to produce influenza VLPs composed of Hemagglutinin (HA), Neuraminidase (NA) and Matrix Protein (M1) and compared to VLPs produced in Sf9 cells through the lens of bioprocessing.

## Materials and methods

VLPs from both systems were characterized using currently available influenza quantification techniques such as Single Radial Immunodiffusion (SRID) assay, Hemagglutination (HA) assay, Negative Staining Electron Microscopy (NSEM) and western blot.

## Results

It was found that VLPs from the HEK 293SF system were present in the culture supernatant in a heterogeneous mixture in terms of particle shape and size. Particles were spherical and also pleomorphic in shape and ranged from sizes of 100-400 nm. Sucrose cushion concentrated samples contained broken particles and a lot of debris. Additionally, it was found that VLPs were associated with the cell pellet after harvest in relatively the same amount as released into the supernatant in the form of unreleased VLPs from NSEM and HA assay analysis. This is possibly due to the sticky nature of the HA protein or from cell clumping during production that worked to trap the VLPs, preventing release into the supernatant. Sf9 cells produced more uniformly shaped VLPs that were spherical in shape, around 100 nm in size and were found to be mainly in the supernatant, not associated with the cell pellet. Sucrose cushion concentrated VLPs contained noticeably less debris than VLPs produced from HEK 293SF cells. It was found that VLP production in Sf9 cells produced 1.5 logs more VLPs than in HEK 293SF cells and had 30× higher HA activity. However, Sf9 VLP samples contained 20× more baculovirus than VLPs, which can contribute to HA activity in both the HA and SRID assays which has to be acknowledged during process development stages. This is the first time to our knowledge that specific production values for influenza VLPs in terms of total particles/ml have been reported.

## Conclusions

From this study, the insect-cell baculovirus system produced a more homogeneous population of VLPs compared to its counterpart in HEK 293SF cells. However, this study also highlights the major problem of baculovirus contamination in the Sf9 system, which requires removal for final vaccine formulations and to help ease the optimization of process production conditions.
